# Stepwise crystallographic visualization of dynamic guest binding in a nanoporous framework†
†Electronic supplementary information (ESI) available: Materials and instrumentations, synthesis and characterization, experimental details, X-ray refinement details, TGA, DRS and FTIR. CCDC 1475220 (**2**), 1475221 (**3**), 1475222 (**4**). Full crystallographic data for the solved structures have been deposited in the Cambridge Crystallographic Data Center. For ESI and crystallographic data in CIF or other electronic format see DOI: 10.1039/c7sc00267j


**DOI:** 10.1039/c7sc00267j

**Published:** 2017-02-13

**Authors:** Gabriel Brunet, Damir A. Safin, Mohammad Z. Aghaji, Koen Robeyns, Ilia Korobkov, Tom K. Woo, Muralee Murugesu

**Affiliations:** a Department of Chemistry and Biomolecular Sciences , University of Ottawa , Ontario K1N 6N5 , Canada . Email: twoo@uottawa.ca ; Email: m.murugesu@uottawa.ca; b Institute of Condensed Matter and Nanosciences , Université Catholique de Louvain , Place L. Pasteur 1 , B-1348 Louvain-la-Neuve , Belgium

## Abstract

Binding sites are at the heart of all host–guest systems, whether biological or chemical. When considering binding sites that form covalent bonds with the guest, we generally envision a single, highly specific binding motif. Through single-crystal X-ray crystallography, the dynamic binding of a guest that displays a variety of covalent binding motifs in a single site of adsorption is directly observed for the first time. The stepwise crystallographic visualization of the incorporation of I_2_ within a porous MOF is presented, wherein the preferred binding motifs throughout the uptake process are identified. The guest I_2_ molecules initially bind with terminal iodide atoms of the framework to form [I_4_]^2–^ units. However, as the adsorption progresses, the I_2_ molecules are observed to form less energetically favorable I_3_^–^ groups with the same framework iodide atoms, thereby allowing for more guest molecules to be chemisorbed. At near saturation, even more binding motifs are observed in the same pores, including both physisorbed and chemisorbed guest molecules. Herein, we present the successful identification of a unique set of host–guest interactions which will drive the improvement of high capacity iodine capture materials.

## Abstract 2

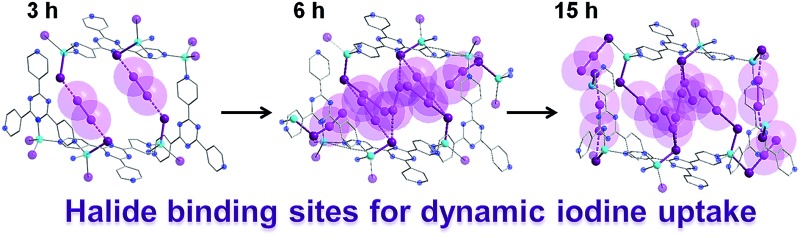
The dynamic uptake behaviour of a gaseous guest has been observed crystallographically, yielding a unique and ever-changing set of host–guest interactions that will drive the improvement of high-capacity iodine capture materials.

## Introduction

Binding sites are ubiquitous in chemical and biological systems, extending from small molecule adsorption in carbon capture materials to complex ligand binding sites in proteins.^1^ When considering binding sites with high selectivity towards a certain substrate, we inherently envision a single binding motif organized by specific host–guest interactions. The nature of these interactions are generally elucidated through spectroscopic methods, however, the direct observation of binding sites through single crystal X-ray diffraction (SCXRD) provides the most indisputable evidence of the binding interactions at the atomic level.^2^ The application of SCXRD is particularly challenging for gas adsorption processes, which are highly fluxional. Only in a few cases has the direct crystallographic observation of adsorption sites in metal–organic frameworks (MOFs) and other nanoporous solids been achieved.^3^ In all cases, each adsorption site only displayed a single binding motif – even when weak physisorption was involved. When the binding is chemisorptive, one would not expect a binding pocket to accommodate a single substrate in more than one way, since covalent interactions tend to be highly specific, directional and strong. To date, the direct observation of a binding site that can accommodate gaseous substrates in a number of different binding modes has yet to be reported. Herein, we present for the first time the dynamic binding of a gaseous guest that displays a variety of covalent binding motifs in a single adsorption site. This remarkable behavior has been directly observed in a stepwise crystallographic fashion, where the chemisorption of I_2_ within a porous MOF exhibits changing covalent bonding motifs in the same pore to accommodate a larger number of gas molecules.

With an increasing interest in the development of gas capture materials, MOFs serve as a new and exciting avenue to explore due to their facile functionalization and high surface areas. The ability to control the framework components has resulted in a steady year-over-year improvement of the uptake capacities and selectivities for various gases of interest.^4^ In particular, the capture and sequestration of highly mobile volatile gasses produced from nuclear fission, such as ^129^I and ^131^I, is becoming an extensive area of research in the field of porous materials.^5^ Such radionuclides pose a significant risk to human health and the environment, therefore, strategies to efficiently store radioactive iodine in a durable waste form must be developed. The present work seeks to provide new insights into the fascinating binding interactions exhibited by I_2_, and hence improve upon the current state-of-the-art radioactive iodine capture methods. Our strategy relies on the use of halide binding sites separated through optimal distances, thereby providing favorable halide–halide interactions between the guest and the host.

While studying materials for the selective uptake of I_2_, we have surveyed porous MOFs with iodide functional groups lining the pores that would act as anchor sites for I_2_ adsorption. One such material that we have examined is the {[(ZnI_2_)_3_(TPT)_2_]·5.5(C_6_H_5_NO_2_)}_*n*_ (**1**) MOF, developed by Biradha *et al.*, that is built from inorganic nodes of ZnI_2_ linked by the 2,4,6-tris(4-pyridyl)-1,3,5-triazine (TPT) ligand.^6^ We found that the material displays a near record breaking I_2_ gravimetric uptake capacity and that there were significant deformations of the framework triggered by the adsorption. Further investigation of the adsorption process by SCXRD allowed for the direct visualization of I_2_ binding in **1**, and the remarkable discovery that the binding motif changes as the adsorption proceeds. Hence, we provide a detailed investigation, through crystallographic and computational methods, of the changes in the binding sites of I_2_ throughout the uptake process.

## Results and discussion

### Inclusion procedure

**Fig. 1:**
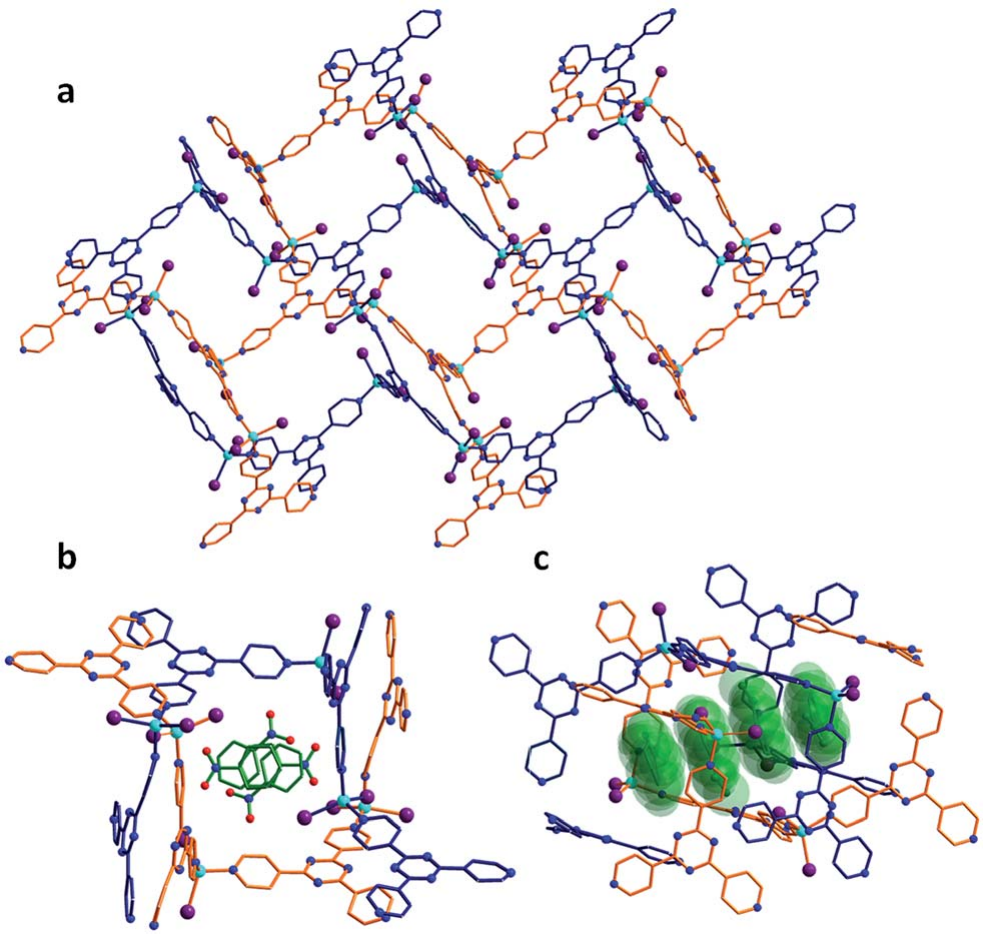
Crystal structure of the as-synthesized MOF **1**. (a) Crystal packing of the empty framework **1** along the *b*-axis, illustrating the doubly-interpenetrated structure. (b) Cross-section of the pores of **1**, which are initially filled with nitrobenzene molecules. (c) Side-view of the continuous channels found in **1** and the nitrobenzene guests displayed in space-filling diagrams.

**Fig. 2:**
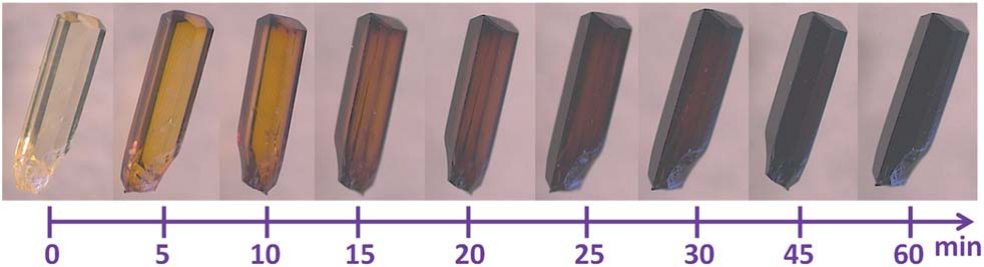
Time-lapsed photographs demonstrating the color change associated with the exposure of single crystals of **1** to I_2_ vapors, as viewed under optical microscope.

The porous MOF **1** exhibits a doubly interpenetrated structure, leading to the formation of continuous channels with pore apertures of 8 × 5 Å^2^ (Fig. 1). As-synthesized single crystals of **1** initially contain nitrobenzene as guest molecules, which interact strongly with one another due to π···π stacking interactions. A detailed description of the structure and properties of **1** have been presented elsewhere.^6^ Upon the exposure of **1** to I_2_ vapors in a closed vessel at room temperature, a pronounced and rapid color change in the single crystals, from colorless to black, could be observed (Fig. 2). We have successfully obtained three crystal structures by varying the time of exposure of **1** to the I_2_ vapors from 3 to 15 h, providing us with vital information on the mechanism by which I_2_ becomes incorporated in the MOF. Exposure times beyond 15 hours resulted in weaker diffraction and eventual loss of crystallinity. Nevertheless, the host framework continues the uptake of I_2_ up to approximately 72 h, after which the MOF becomes saturated (*vide infra*). The degree of I_2_ encapsulation will likely depend on the size of the crystals,^7^ and therefore, efforts were made to select single crystals of approximately equal size for the SCXRD experiments.

### Formation of [I_4_]^2–^ bridges

**Fig. 3:**
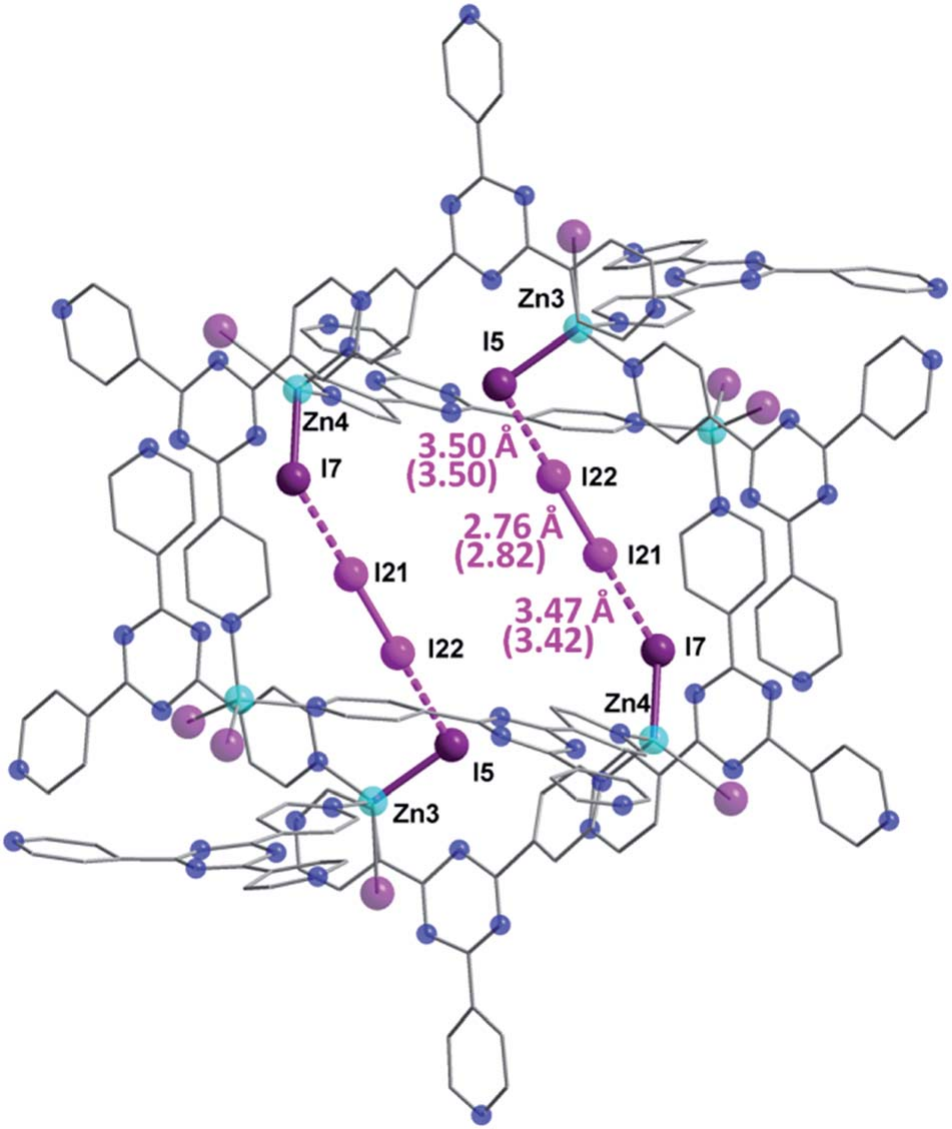
Molecular structure of a single pore of **2**, emphasizing the initial preferred binding mode of the I_2_ guest which forms strong halogen–halogen interactions with the iodide ions of the framework, effectively forming an [I_4_]^2–^ unit. Selected geometric parameters are shown, with DFT computed values given in parentheses. Guest iodine atoms are shown in a lighter shade of purple than the iodide atoms belonging to the host framework. Hydrogen and disordered atoms are omitted for clarity.

The first SCXRD structure, obtained after 3 h of **1** being immersed in vapors of I_2_, revealed large peaks of electron density within the pores, which were identified as multiple I_2_ molecules, giving the first intermediate {[(ZnI_2_)_6_(TPT)_4_]·1.3(I_2_)·8.65(C_6_H_5_NO_2_)}_*n*_ (**2**). It is important to note that the chemical formulas of the iodine-containing MOFs described herein highlight the number of adsorbed I_2_ molecules, rather than the newly formed iodine species. The crystal system remains in the monoclinic family, however the space group changes from *C*2/*c* in **1** to *P*2_1_/*c* in **2**. Moreover, analysis of the crystal structure indicates that there are approximately 5.2 I_2_ molecules per unit cell, resulting in an iodine content of ∼7.2 wt% (excluding the framework iodide atoms). With an increase in the iodine content, we conversely observe a decrease in the amount of nitrobenzene molecules from 44 to 34.6 per unit cell. This exchange process, where the nitrobenzene molecules are readily replaced by the encroaching I_2_, provides evidence that the interactions formed by the I_2_ species are more favorable than the π···π stacking interactions of the nitrobenzene guests (Fig. 1c). Careful analysis of the crystal structure of **2** reveals that the highest occupied I_2_ guest (I21–I22), and thus the most favourable initial binding site, is positioned between two iodide atoms (I5 and I7) originating from the ZnI_2_ building units of the host structure (Fig. 3).

The bond distance between the I_2_ molecule (I21–I22) and framework iodide atoms are found to be 3.50 and 3.47 Å, while the bond length in the guest I_2_ elongates from a molecular value of 2.67 Å to 2.76 Å upon formation of **2** (Table S1†). This type of M–I···I–I···I–M linkage has been previously reported in a handful of discrete compounds and polymeric chains,^8^ wherein the four iodine atoms are characterized as forming a [I_4_]^2–^ unit. However, this represents the first MOF structure containing this type of unusual bridging unit. The [I_4_]^2–^ fragment is approximately linear with the largest deviation coming from the I5···I22–I21 angle (∼173.0°). It is also important to note that three other distinct I_2_ molecules have been identified in **2**, with occupancies ranging from 4.6–7.9% (Table S1†). These sparsely occupied guest molecules reveal the subsequent binding sites, which will dominate the uptake behaviour upon further I_2_ exposure, in the form of I_3_^–^ anions (*vide infra*). Additionally, a second [I_4_]^2–^ unit can also be located in an adjacent pore to the first [I_4_]^2–^ linkage, however, its occupancy is 15.5% due to the presence of an overlapping nitrobenzene solvent molecule.

In order to provide further insight into the nature of the I_2_ binding in **2**, we have employed dispersion corrected periodic DFT calculations. Geometry optimization of **2** starting from the crystal structure gives a computed structure in excellent agreement with the SCXRD structure (Fig. S1†), with the I–I bond distances deviating no more than 0.06 Å (parenthetic values in Fig. 3). To investigate the degree of covalency in the I^–^···I_2_ interactions, Wiberg bond orders were calculated (Table S1†). The dual end-on bridging interaction of the I_2_ guests with the terminal iodides of the framework gave bond orders of 0.20 and 0.24, for I5···I22 and I7···I21, respectively. By comparison, the I21–I22 pair has a bond order of 0.78 in **2**. These results, combined with the calculated I_2_ bonding energy of 26.2 kcal mol^–1^, indicate that the I_2_ adsorption in **2** is chemisorptive in nature.

### Increased I_2_ uptake and replacement of [I_4_]^2–^ bridges

The same iodine vapor diffusion strategy was employed to obtain the second intermediate structure {[(ZnI_2_)_6_(TPT)_4_]·2.51(I_2_)·3(C_6_H_5_NO_2_)}_*n*_ (**3**), after 6 h of exposure time to the I_2_ vapors. The dark brown crystals remained in the *P*2_1_/*c* space group (Table S2†), yet yielded more electron density within the channels of the MOF, assigned to I_2_ molecules. The host framework maintains the structural architecture and topology of **1**, however, we can observe a further decrease in the unit cell volume going from **2** to **3** (∼0.8%). The encapsulation of additional I_2_ guests when going from **2** to **3** is accompanied by a further reduction in nitrobenzene solvent molecules, down from 34.6 to 12 molecules per unit cell following 6 h of exposure time to I_2_ vapors. Accordingly, the iodine guest content increased from 7.2 wt% to 15.3 wt%, yielding ∼10 I_2_ molecules per unit cell. This drastic increase in merely 3 h of additional exposure time emphasizes the potential of this framework for the rapid capture and sequestration of I_2_.

**Fig. 4:**
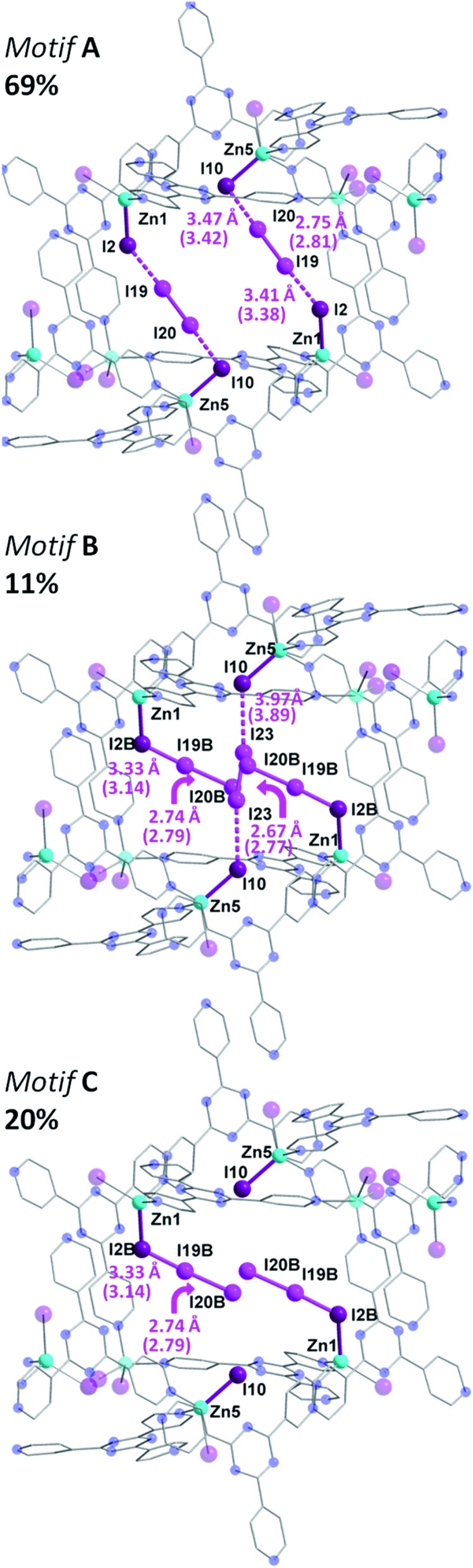
Molecular structure of a single pore of **3**, illustrating the disordered and partially occupied I_2_ guests. In motif **A** (top), there is the same [I_4_]^2–^ unit as in **2**, while in motif **B** (middle), there are chemisorbed I_3_^–^ group along with an I_2_ molecule forming an [I_4_]^2–^ unit. Motif **C** is also shown (bottom). Selected geometric parameters are displayed with DFT computed values given in parenthesis. Guest iodine atoms are shown in a lighter shade of purple than the iodide atoms belonging to the host framework. Hydrogen and additional disordered atoms are omitted for clarity.

In comparison to the principal I_2_ binding motif observed in **2**, careful analysis of the crystal structure of **3** reveals eight distinct I_2_ binding positions with partial occupancies. More interestingly, I_2_ molecules occupy the same crystallographically equivalent pore in three distinct manners, with occupancies of 69, 20 and 11% (Fig. 4). The high occupancy (69%) binding motif, referred as motif **A**, is identical to the I_2_ adsorption observed in **2**. The I···I distances and computed bond orders (Tables S3 and S4†) are very similar as those in **2** and we can therefore characterize the adsorption as chemisorptive. The lower occupancy binding motif (11%), referred as motif **B**, not only accommodates an additional I_2_ molecule compared to motif **A**, but does so in a completely different manner even though the same atoms of the MOF framework form bonds with the I_2_ guests. In motif **A**, two I_2_ molecules each form [I_4_]^2–^ moieties with atoms I2 and I10 of the framework. In motif **B**, each of the two I2B framework atoms instead form an I_3_^–^ group with the I_2_ guests (I19B–I20B), and a third I_2_ molecule forms an [I_4_]^2–^ moiety with the two I10 atoms (I23). It is noteworthy that the I_3_^–^ and [I_4_]^2–^ fragments in motif **B** are non-interacting with a shortest I···I separation of 4.48 Å.

The formation of the triiodide group in motif **B** suggests that the I_2_ molecule is chemisorbed. The bond distance between the framework I2B atom (which participates in I_3_^–^ formation, whereas I2 forms the [I_4_]^2–^ unit) and the I19B atom of the adsorbed I_2_ is 3.33 Å, consistent with the parameters of an I_3_^–^ moiety.^9^ The DFT calculated I2B–I19B bond order is 0.45, while the binding energy was determined to be 21.6 kcal mol^–1^. These metrics are all consistent with chemisorption. The SCXRD data suggests that the terminal I19B–I20B bond length of the I_3_^–^ moiety in motif **B** is 2.74 Å, and is in strong agreement with the DFT optimized geometry of motif **B**, which gives a I19B–I20B bond length of 2.79 Å.

In addition to the two I_2_ molecules that adsorb to form triiodide groups, motif **B** accommodates a third I_2_ molecule that forms a [I_4_]^2–^ unit which bisects the pore. DFT calculations also point towards the bonding being chemisorptive. For example, the calculated binding energy was determined to be 20.2 kcal mol^–1^, which is slightly lower than the 21.6 kcal mol^–1^ value for the triiodide formation. Furthermore, using the DFT optimized structure, the I10–I23 bond order was found to be 0.15 (Table S3†). It is interesting to note that four additional I_2_ binding positions are observed in **3**, but are located in a different pore (Fig. S2†). This pore accommodates three I_2_ guest molecules. A more detailed discussion of the bonding in this second pore is given in the ESI.† While motif **A** and motif **B** account for the I_2_ guests in 69 and 11% occupancies, respectively, the remaining 20% is accounted by I19B–I20B, which, as previously stated, forms an I_3_^–^ unit with I2B and yields motif **C** (Fig. 4). This third binding motif is identical to motif **B**, without the bisecting I23–I23 molecule that forms the [I_4_]^2–^ linkage. As such, the total occupancy of I19B–I20B is 31%, through the combination of motif **B** and motif **C**. Thus, motif **B**, which encapsulates I23, can only be present when motif **A** is absent, clearly reinforcing the notion of varying bonding motifs with an increase in I_2_ uptake.

This structural intermediate provides an excellent model to deduce the mechanistic pathway of I_2_ inclusion. Indeed, each terminal I^–^ ion of the framework serves as a binding site for the I_2_ guests and display a variety of binding modes. The driving factor towards observing this unprecedented behavior is the energetic stability gained by the incorporation of increasing amounts of I_2_ molecules within the MOF. Bonding motif **A**, wherein the I_2_ molecules bind to form [I_4_]^2–^ moieties, has the strongest calculated binding energy of 26.6 kcal mol^–1^ per I_2_ molecule. However, this motif only accommodates two I_2_ molecules. In order to accommodate a third I_2_ molecule, two [I_4_]^2–^ units can rearrange to form less energetically favorable I_3_^–^ groups. Evaluation of the bonding energy of isolated I_3_^–^ groups, without the third I_2_ molecule, yields a bonding energy of 21.2 kcal mol^–1^. Thus, while the I_3_^–^ groups form stronger covalent interactions with the terminal I^–^ ions of the framework, compared to the [I_4_]^2–^ units, their energetic configuration is less favorable. Overall, however, the energy of motif **B**, with three I_2_ molecules adsorbed, is 9.0 kcal mol^–1^ more stable than the energy of motif **A**, containing two adsorbed molecules, plus the energy of a free I_2_ molecule.

### Continued sequestration of I_2_

**Fig. 5:**
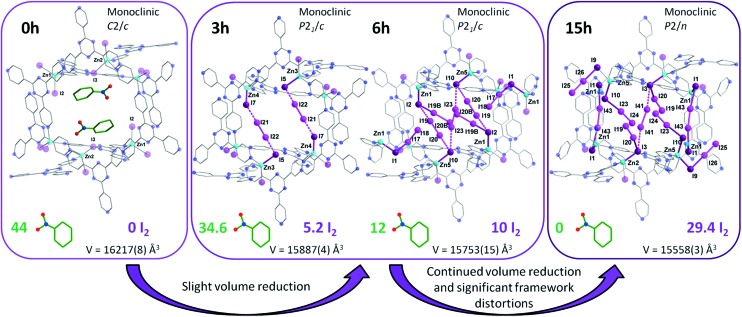
X-ray crystal structure of a single pore of **1** viewed along its channel direction. Evolution of the guests, where nitrobenzene solvent molecules are sequentially exchanged for I_2_ molecules, are illustrated for the same pore following 3, 6 and 15 h of exposure time to I_2_ vapors. The amount of localized guest molecules contained per unit cell are listed, along with changes in the space group and unit cell volumes for compounds **1–4**. Guest iodine atoms are shown in a lighter shade of purple than the iodine atoms belonging to the host framework. Hydrogen and additional disordered atoms are omitted for clarity.

Following multiple attempts to measure the SCXRD data of **1** saturated with I_2_ it was determined that 15 h of exposure time to I_2_ vapors was the limit for retaining adequate crystallinity. Hence, we present the single crystal structure of {[(ZnI_2_)_6_(TPT)_4_]·7.34(I_2_)}_*n*_ (**4**), with the best of our refinement results. Intermediate **4** was refined in the monoclinic *P*2/*n* space group and further continues the trend of decreasing unit cell volumes with I_2_ uptake, reflecting the flexible nature of the MOF. It is important to note that while the agreement factors of the refinement are higher than in **2** and **3**, due to the increase in partially occupied and positionally disordered guest molecules, the crystal structure of **4** provides invaluable structural insights regarding the encapsulation of I_2_. Notably, we can observe the absence of nitrobenzene solvent molecules and their exclusive replacement by I_2_. The unit cell now comprises approximately ∼29.4 localized I_2_ guest molecules, a radical increase of 198% going from **3** to **4**. Thus, the iodine guest content is ∼37.2 wt%. In other words, 1 g of the empty framework of **1** can uptake around 0.59 g of I_2_ after 15 h which at this stage outperforms state-of-the-art zeolites, and is comparable to other porous MOFs.^10^ These results are in good agreement with the TGA measurements performed on single crystals of **4**, giving an iodine guest content of 39.8% and an uptake of I_2_ of 0.66 g g^–1^ (Fig. S3†). This drastic increase in I_2_ inclusion from 9 additional hours of I_2_ vapor exposure results in a significant distortion of the MOF framework. The changes in the pores from the as-synthesized MOF **1**, to the structure of **2** and **3** after 3 and 6 h of exposure time to I_2_ vapors, respectively, followed by the 15 h structure **4** are illustrated in Fig. 5. We can surmise that these distortions, which allow for a greater intake of I_2_, are a contributing factor in the loss of crystallinity of the single crystals. The connectivity of the framework remains the same, however the pyridine moieties, as well as the central triazine rings of the TPT ligand, are less planar and adopt a more twisted conformation as evidenced by the large thermal parameters. We can hypothesize that these groups exhibit some freedom of rotation, therefore permitting the entry and accommodation of a larger number of I_2_ guests within the same pore or channel. Structural analysis of **4** reveals that the initial [I_4_]^2–^ unit found in **2** and **3** has been replaced with several partially occupied physisorbed and chemisorbed I_2_ molecules, also supporting the previous assessment of reorienting the [I_4_]^2–^ unit to accommodate a larger number of I_2_ guests. While **3** displayed a significant increase in electron density assigned to I_2_, their occupancies remained relatively low, whereas **4** sees the incorporation of an even larger amount of I_2_ combined with higher occupancies (Table S5†). Chemisorption remains the preferred method of adsorption, with 13 individually refined I_2_ molecules covalently bound to I^–^ ions of the framework. Furthermore, several [I_4_]^2–^ units can also be identified in a different pore and in different emplacements than in the structures of **2** and **3** (Fig. S4†). It is worthwhile to mention that several I_2_ guest molecules are positionally disordered, such as I23–I24, which forms an I_3_^–^ moiety with I10, undergoes a precession movement leading to a disordered I24 atom. The remaining I_3_^–^ groups give distances and angles within a reasonable range to those found in the literature.^11^ The I_2_ guests involved in the formation of [I_4_]^2–^ units, are weakly halogen bonded to terminal iodide ions of the framework, as displayed by elongated I^–^···I_2_ distances (Table S5†). The orientation of the guest along the two terminal I^–^ ions strongly suggests the formation of the Zn–I···I–I···I–Zn linkage, in similar fashion to the [I_4_]^2–^ unit described in **2**. To complete the analysis of the guests, two physisorbed I_2_ molecules can also be identified. It is remarkable that even with the inclusion of all these I_2_ molecules, **4** is still able to encapsulate additional I_2_ molecules. This was confirmed by the TGA curve of a sample of **1** exposed to I_2_ vapors over a period of 72 h, giving a weight loss of ∼63.4% (Fig. S3†). Hence, one gram of **1** has the potential of loading an excess of 1.73 g of I_2_ at room temperature, making it an exceptional material for the capture of iodine. This value is comparable with the highest I_2_ uptake capacity reported for a MOF, namely Cu-BTC (1.75 g g^–1^), which was measured at 75 °C.^12^ This enormous uptake of I_2_ in **1** can be attributed to a number of factors, including favorable guest–host interactions, framework flexibility, and high porosity (∼60%). More specifically, **1** offers strong sites of adsorption for I_2_ through the terminal iodide ions of the framework as well as favorable π–halogen interactions with the TPT ligand.

### Stability and release kinetics

**Fig. 6:**
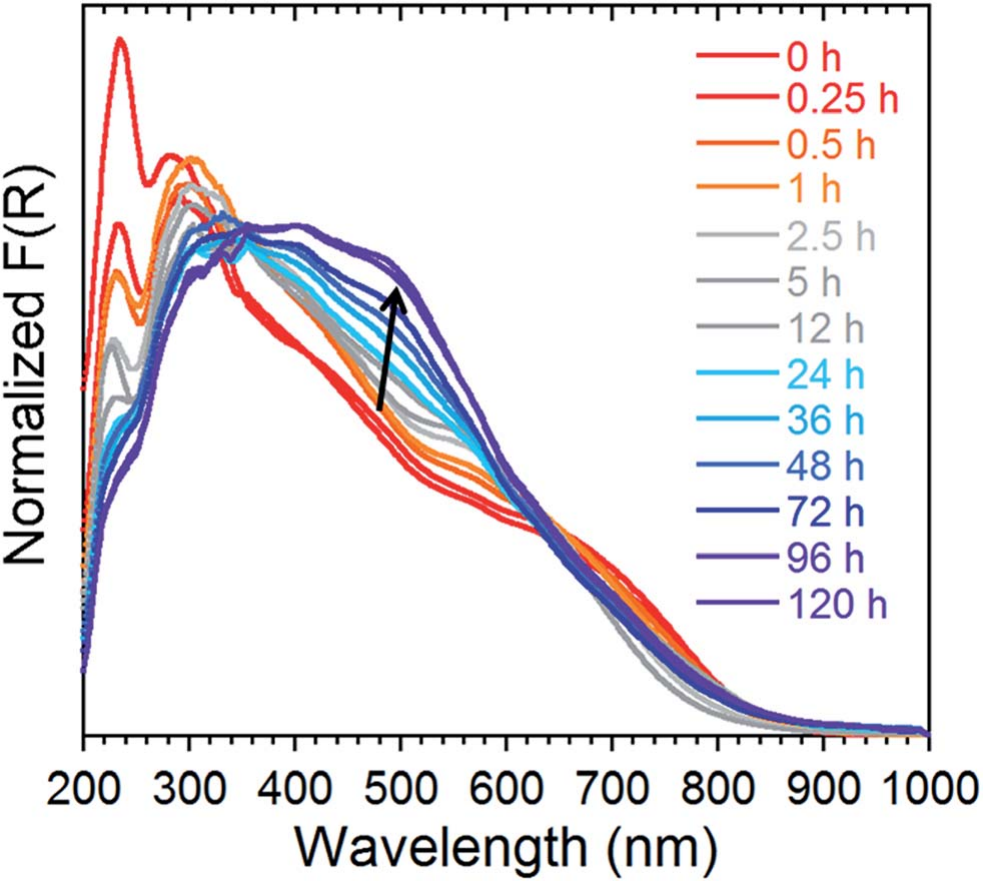
Normalized Kubelka–Munk spectra of **1** saturated with I_2_ followed over time at ambient conditions for a period of 120 h.

In order to probe the release kinetics of iodine, we performed time-dependent diffuse reflectance spectroscopy (DRS) measurements on compound **1** once it was completely saturated by I_2_. The Kubelka–Munk spectra of TPT, ZnI_2_, I_2_ and **1** were first collected to examine the origin of the bands (Fig. S5†). Afterwards, single crystals of **1**, saturated with I_2_, were measured through DRS over a period of 120 h at ambient conditions (Fig. 6). The time-lapsed Kubelka–Munk spectra reveal that stabilization of the compound occurs after approximately four days. This stabilized compound exhibits similar electronic transitions as **1** saturated with I_2_, providing strong evidence that iodine remains a major component of the MOF. In principle, we can envision that the more weakly encapsulated I_2_ guests (*i.e.* physisorbed) are more susceptible of being evacuated, while the chemisorbed iodide molecules would remain part of the framework and require harsher conditions for their removal. A detailed examination of the time-dependent DRS measurements shows a decrease in the intensity of the bands in the UV region, combined with an increase in the intensity and a blue-shift of the shoulders in the visible range. This may be explained by the repulsive interactions of the I_2_ molecules. The stabilized compound (after 96 h), is weakly diffracting, and therefore, cannot be examined by conventional crystallographic methods. This compound was, however, investigated by FTIR, revealing intense and broad bands at approximately 670, 1640 and 3350 cm^–1^, which is characteristic of the vibrations for H_2_O (Fig. S6†). Thus, if **1** is saturated by I_2_ and subsequently stored at ambient conditions, we can expect evaporation of the physisorbed I_2_ followed by incorporation of water molecules. Nevertheless, the DRS results demonstrate the potential of **1** for the irreversible capture of radioactive I_2_, through a number of strong chemisorption sites.

## Concluding remarks

The direct single crystal observation of gaseous substrate binding is exceedingly rare, due to the difficulty in retaining adequate crystallinity following guest encapsulation and the lability of gases. In this work, we have successfully elucidated the process by which gaseous I_2_ is systematically incorporated in the cavities of a highly porous MOF, through the use of stepwise crystallographic experiments and computation. Remarkably, the guest I_2_ molecules are found to adopt multiple covalent bonding motifs in the same adsorption site, even with the same framework atoms, depending on the percentage of uptake. This is unique in that covalent bonding tends to be highly directional, specific and strong, thereby commanding a single binding motif organized by these interactions. The participation of both physisorption and chemisorption in the uptake of gaseous guests is another fascinating feature of the MOF, since chemisorption is generally associated with frameworks exhibiting open metal sites that can bind guest molecules.^13^ This illustrates the optimization potential of MOFs, where, notably, chemisorption can be obtained without open metal sites, leading to higher chemical stabilities. Hence, we envision that this study will aid in the rational design of MOFs for the capture of gaseous fission products, through a fundamental understanding of the dynamics and site selection of gaseous substrates. The design strategy involving anchor sites based on halide–halide interactions is highly promising for the enhancement of radioactive iodine capture materials.
