# Supplementary material for: Stepwise crystallographic visualization of dynamic guest binding in a nanoporous framework
Source: Chem Sci. 2017 Feb 13;8(4):3171–7. doi: 10.1039/c7sc00267j (PMC5465547; doi:10.1039/c7sc00267j)
Supplement: Supplementary file 1 [file SC-008-C7SC00267J-s001.pdf]

Electronic Supplementary Material (ESI) for Chemical Science.
This journal is © The Royal Society of Chemistry 2017

Supporting Information

Stepwise Crystallographic Visualization of Dynamic Guest Binding in a Nanoporous Framework

Gabriel Brunet,[a] Damir A. Safin,[a] Mohammad Z. Aghaji,[a] Koen Robeyns,[b] Ilia Korobkov,[a] Tom K. Woo,[a]* and Muralee Murugesu[a]*

[a] Department of Chemistry and Biomolecular Sciences, University of Ottawa, Ontario, Canada, K1N 6N5.

[b] Institute of Condensed Matter and Nanosciences, Université Catholique de Louvain, Place L. Pasteur 1, B-1348 Louvain-la-Neuve, Belgium.

Corresponding Authors

*e-mail: twoo@uottawa.ca; m.murugesu@uottawa.ca

This PDF file includes:

Supplementary Materials and Methods

Supplementary Figures 1-7

Supplementary Tables 1-5

Materials, instrumentation and synthesis

General considerations

Starting materials and solvents were purchased and used without further purification from commercial suppliers (Strem Chemicals, Sigma-Alrich and Fisher Scientific).

Physical measurements

Infrared spectra were recorded with a Varian 640 FTIR spectrometer equipped with an ATR in the 500–4000 cm^{-1} range. Diffuse Reflectance spectra were measured with a Varian Cary-100 spectrophotometer using polytetrafluoroethylene (PTFE) as a reference. Kubelka-Munk spectra were normalized to allow meaningful comparisons. Thermogravimetric analysis (TGA) data were recorded using a Q5000 IR TGA instrument at a heating rate of 5 °C/min between room temperature and 1000 °C , under a constant flow of nitrogen (25 mL/min).

Synthesis

The TPT ligand and compound **1**, $\{[(ZnI_2)_3(TPT)_2]\cdot 5.5(C_6H_5NO_2)\}_n$, were synthesized according to previously reported methods.[1,2] Single crystals of **1** were carefully selected under optical microscope and inserted in a 5 mL vial. This vial was subsequently placed in a 40 mL vial containing ~100 mg of iodine pellets, which was then capped and sealed with Parafilm. Following 3, 6 and 15 hours of exposure time to the iodine vapors, the crystals were then immediately characterized yielding compounds **2**, **3** and **4**, respectively.

Single-crystal data collection

Isolated single-crystals were analyzed on a Bruker-AXS KAPPA diffractometer equipped with a sealed Mo tube source (λ = 0.71073 Å) APEX II CCD detector. Full crystallographic data for the solved structures have been deposited in the Cambridge Crystallographic Data Center with CCDC numbers 1475220 (**2**), 1475221 (**3**), 1475222 (**4**).

Data collection results for compounds **2**–**4** represent the best data sets obtained in several trials for each sample. By visually inspecting single-crystals of **1** exposed to iodine vapors under optical microscope, the appearance of a considerable amount of crystal defects are observed. This can be rationalized by assuming that the interaction of the MOF with iodine vapors not only leads to the diffusion of iodine inside the crystal (resulting in a replacement of nitrobenzene solvent molecules), but also leads to a change in the symmetry and unit cell parameters of the crystal packing, essentially causing a chemically induced phase transition. Hence, even crystals that were deemed transparent contained several closely oriented domains upon X-ray investigation. In addition to crystal fragmentation, it could be easily suggested that due to the "surface to center" diffusion of iodine it is possible to have an unevenly radial distribution of I_2 molecules throughout the crystal. Being aware of the abovementioned problems, many attempts were made to record and process the data as a multiple domains twin of the same phase or even as multiple phase conglomerates. Unfortunately, multi-domain refinement did not yield satisfactory results, some small twin domains were identified by the twinrotmat procedure in Platon during crystal refinement and the final refinement

cycles were performed against a HKLF5 formatted reflection file, representing 1 or more additional small twin domains.

X-ray refinement of compound 2

Diffraction data for the crystal of compound **2** were collected to 0.75 Å resolution, however, a data cut-off of 0.83 Å was imposed during the refinement, above which the refinement statistics became significantly worse. The structural model of **2** contains six ZnI_2 fragments interconnected by 4 TPT ligands as in the starting MOF **1**. Inside the cavities of the MOF, it was possible to locate and successfully model five fully occupied molecules of nitrobenzene along with 5 partially occupied nitrobenzenes (95%, 93%, 84%, 49% and 42% occupancies). Also one fully occupied I_2 moiety and 4 partially occupied I_2 molecules, which are found on the locations of the partially occupied nitrobenzene molecules. In order to maintain acceptable molecular geometries for the nitrobenzene solvent molecules, the benzene ring was constrained to an ideal hexagon and similarity restraints were set up, using a well-defined fully occupied nitrobenzene molecule as reference. All non-hydrogen atoms were refined anisotropically, several restraints on the thermal ellipsoids were necessary to let the refinement converge with proper thermal displacement factors. Significant disorder was also observed for half of the ZnI_2 moieties, for Zn3 and Zn4 two disordered sites were defined, for Zn2 even 3. For the minor components of the ZnI_2 disorder hard thermal motion constrains (EADP) were applied with the major component as reference.

Although a significant number of solvent / I_2 molecules were located, 2 large cavities were found in the unit cell, where no discrete molecules could be located, these cavities

(2x 419 Å^3) were treated by the squeeze procedure in Platon[3] where 138e$^-$ could be located, and added as partial structure factors during subsequent refinement cycles.

X-ray refinement of compound 3

Diffraction data for the crystal of compound **3** were collected to 0.75 Å resolution, however, a data cut-off of 0.83 Å was imposed during the refinement, above which the refinement statistics became significantly worse. The asymmetric unit for this crystallographic model of **3** consists of six ZnI_2 nodes connected with 4 TPT ligands. Inside the pores or channels of the MOF, it was possible to locate one I_2 molecule, disordered over two sites (I19-I20, 69/31occupancy), and 7 more partially occupied I_2 fragments as well as 3 fully occupied nitrobenzene solvent molecules. All the fragments of the structural model are located in the general positions except on iodine fragment I23–I23A that is located on a crystallographic inversion center. All non-hydrogen atoms were refined anisotropically in combination with a general rigid bond restraint on all atoms. Some thermal ellipsoids of carbon atoms and one disordered nitrobenzene molecule were restraint to approximate isothermal behaviour. All nitrobenzene molecules were refined to be geometrically the same. Following the refinement of the core, many relatively large residual electron density peaks were discovered in the cavities of the MOF structure. Both high intensities and relative locations of these peaks were suggestive to the presence of atoms that are heavier than any second raw elements. Those peaks were successfully modeled as partially occupied I_2 fragments.

Although a significant number of solvent / I_2 molecules were located, 2 large cavities were found in the unit cell, where no discrete molecules could be located, these cavities

(2x 1833 Å^3) were treated by the squeeze procedure in Platon[3] where 575e$^-$ could be located, and added as partial structure factors during subsequent refinement cycles.

X-ray refinement of compound 4

Diffraction data for the crystal of compound **4** were collected to 0.85 Å resolution, however, a data cut-off of 0.93 Å was imposed during the refinement, above which the refinement statistics became significantly worse. The asymmetric unit for the crystallographic model of **4** consists of six ZnI_2 nodes connected with 4 TPT ligands. Inside the pores or channels of the MOF, it was possible to locate 20 partially occupied I_2 fragments. No solvent molecules were found in the cavities of compound **4**. All non-hydrogen atoms were refined anisotropically. Four of the 6 ZnI_2 moieties were found to be disordered over 2 sites with refined occupancies. The minor components were constraint to have identical thermal displacement factors as the major component, and a similarity restraint was imposed to retain the correct geometry. A general rigid bond restraint was applied to all non-hydrogen atoms, in combination with a general restraint to approximate isothermal behaviour of the thermal factors.

All I_2 molecules were partially occupied (occupancies were allowed to be refined) and restrained to have the same bond distance. It was discovered that most of these moieties are independent from each other with exception of four I_2 fragments. More specifically, they appear to be located in the same spacial area and therefore were interpreted as positionally disordered moieties (second fragment denoted as B). Moreover, three I_2 fragments were located on inversion centra.

Although a significant number of solvent / I_2 molecules were located, 2 large cavities were found in the unit cell, where no discrete molecules could be located, these cavities

(2x 686 Å^3 , 1x1544 Å^3) were treated by the squeeze procedure in Platon[3] where 286e$^-$ and 493 e$^-$, respectively, could be located, and added as partial structure factors during subsequent refinement cycles.

For all three compounds, the positions of the hydrogen atoms were calculated based on the geometry of related non-hydrogen atoms. All hydrogen atoms were treated as idealized contributions during the refinement. SHELXL2014/7 was used for data refinement.[4]

General Computational Details

All electronic and structural calculations were performed using periodic density functional theory (DFT) calculations, carried out in the Vienna ab initio simulation package (VASP).[5] The PBE exchange-correlation functional[6] and PAW[7,8] potentials were used with a plane wave cut-off of 520 eV with a convergence set to a change of 2×10^{-5} eV/Å. The semi-empirical dispersion potential correction of Grimme[9] was used for the energy and force calculations with the default parameters for the PBE functional. Gamma-point was used to sample the Brillouin zone. Computed geometries and energies reported were calculated using the periodic unit cell taken from the XRD structure. The positions of the hydrogen and iodine atoms belonging to the framework, as well as the guest molecules, were allowed to fully relax, while the remaining atoms of the framework and cell vectors were fixed to the experimental XRD positions.

Reported bond orders were calculated using a cluster model of the material as shown in Figure S1 for **2** and **3**. The geometry used for the bond order calculations were taken from the equivalent optimized structure from the periodic DFT calculations. For

these calculations, the PBE exchange-correlation functional was used with the def2-TZVP basis set within the Gaussian ’09 package.[10] The NBO6.0 software[11] was used to evaluate the bond orders from the single-point Gaussian calculations.

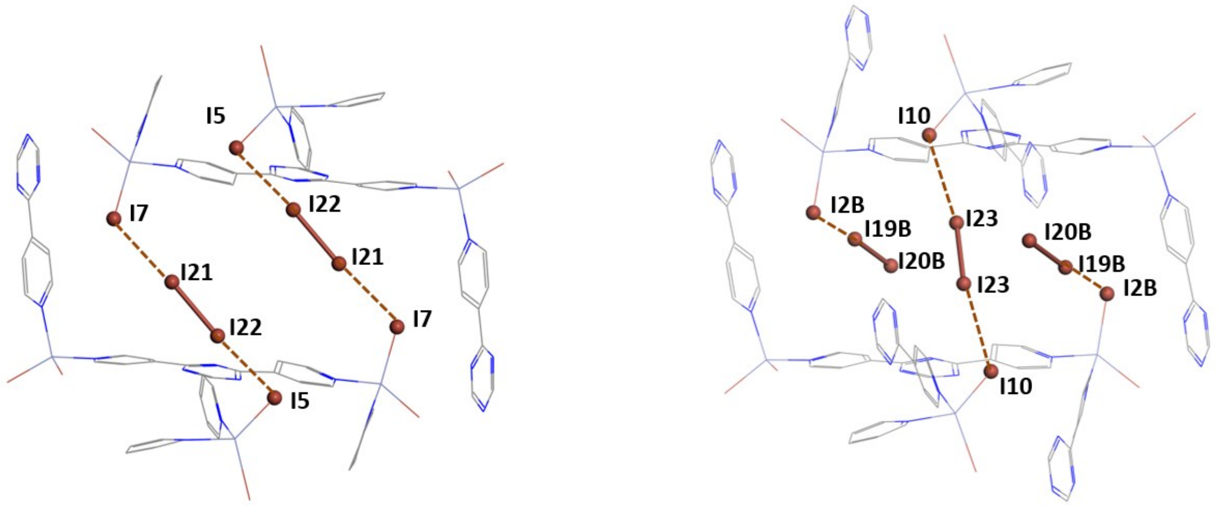

Figure S1. Cluster model used to calculate the bond orders of structures **2** (left) and **3** *motif* **B** (right).

Computational details for compound 2

The initial structures for the DFT study of I_2 binding in a single pore of compound **2** was taken the XRD structure. All solvent molecules and disorder were removed from the structures, along with the I_2 molecules from all pores except one. As discussed in the main text, the resulting geometric parameters were found to be in good agreement with the experimental XRD structure. Calculations were also performed where the I_2 guests were manually displaced to be perpendicular to the terminal I^- ions in the initial structures. Geometry optimization of these structures resulted in the realignment of the I_2 guests back to the position found in **2**. The reported DFT binding energies of I_2 were

calculated using an empty framework structure where all solvent and all guest molecules were removed. Only the hydrogen atoms of the empty MOF were geometry optimized, while all other atoms and the cell vectors were fixed. The binding energy was then calculated using the following equation:

$$E_{binding\ energy} = E_{MOF\ +\ guest(s)} - E_{MOF} - E_{guest(s)}$$

where the energy of the guests were determined from a geometry optimization of a single I_2 molecule placed in an empty simulation cell with the same cell vectors as the MOF. For the energy of the MOF with guests ($E_{MOF+guest(s)}$) two I_2 atoms were placed in the pore as shown in Figure 3 of the main text and optimized. This gave a binding energy of 52.4 kcal/mol (or 26.2 kcal/mol per I_2 molecule).

Table S1. Assignment, occupancy, computational and experimental bond distances, and Wiberg bond orders of iodine guests in compound **2**.

Iodine guest	Occupancy	Assignment	Bond distances (Computational)	Bond distances (Experimental)	Bond order
I21–I22	1	I_4^{2-}<br>(I7–I21–I22–I5)	I7–I21: 3.42 Å<br>I21–I22: 2.82 Å<br>I22–I5: 3.50 Å	I7–I21: 3.47 Å<br>I21–I22: 2.76 Å<br>I22–I5: 3.50 Å	0.24<br>0.78<br>0.20
I23–I24	0.069	I_3^-<br>(I10–I23–I24)		I10–I23: 3.10 Å<br>I23–I24: 2.76 Å	
I25–I26	0.046	I_3^-<br>(I6–I25–I26)		I6–I25: 2.93 Å<br>I23–I24: 2.76 Å	
I27–I28	0.155	I_4^{2-}<br>(I11–I27–I28–I4)		I11–I27: 3.54 Å<br>I27–I28: 2.75 Å<br>I28–I4: 4.20 Å*	
I29–I30	0.079	I_3^-<br>(I6–I25–I26)		I7–I29: 2.73 Å*<br>I29–I30: 2.75 Å	

* Denoted bond distances are the average value for disordered atoms.

Computational details for the exchange of nitrobenzene for I_2

To investigate the exchange of nitrobenzene for I_2, DFT calculations were carried out on a single pore of compound **2**, with the structure taken from X-ray diffraction (XRD). All I_2 guests and disorder were removed from the XRD structure, along with the solvents from all pores except one. Using a similar procedure to calculate the binding energy of I_2 in compound **2**, the binding energy of nitrobenzene in a single pore of compound **2** was calculated to be 9.2 kcal/mol, which is less than the binding energy of an I_2 guest in an equivalent structure (26.2 kcal/mol). This evidence supports the exchange of nitrobenzene for I_2.

Single-Crystal X-ray data

Table S2. Summary of the crystal structure data and refinement for compounds **1**–**4**.

**Complex**	**1**	**2**	**3**	**4**
Formula	$C_{69}H_{51.5}I_6N_{17.5}O_{11}Zn_3$	$C_{123.9}H_{91.25}I_{14.7}N_{32.65}O_{17.3}Zn_6$	$C_{90}H_{63}I_{17.02}N_{27}O_6Zn_6$	$C_{72}H_{48}I_{26.68}N_{24}Zn_6$
FW, g mol^{-1}	2259.29	4572.24	4171.36	5027.89
Crystal system	monoclinic	Monoclinic	monoclinic	monoclinic
Space group	*C*2/*c*	*P*2$_1$/*c*	*P*2$_1$/*c*	*P*2/*n*
T, K	193(2)	200(2)	200(2)	200(2)
a, Å	36.079(10)	35.143(5)	35.047(15)	30.839(4)
b, Å	14.978(4)	15.039(2)	15.0048(8)	14.9128(18)
c, Å	30.734(9)	30.680(4)	30.5654(15)	34.344(4)
α, º	90	90	90	90
β, º	102.470(5)	101.549(4)	101.464(2)	99.929(7)
γ, º	90	90	90	90
V, Å^3	16 217(8)	15 887(4)	15 752.9(15)	15 558(3)
Z	8	4	4	4
ρ_{calcd}, g cm^{-3}	1.851	1.686	1.671	1.855
μ (Mo, Kα), mm^{-1}	3.231	3.663	3.957	5.171
reflns collected	14 243	22 219	38 776	19 152
*R*1, *w*R2 (I > 2 σ (*I*))^a	0.0794, 0.2316	0.1471, 0.3200	0.1305, 0.2766	0.1883, 0.3765
*R*1, *w*R2 (all data)	0.1278, 0.2697	0.1727, 0.3311	0.1391, 0.2810	0.2238, 0.3948

^{a} $R_1 = (||F_o| - |F_c||)/|F_o|$; $wR_2 = \{[w(F_o^2 - F_c^2)^2]/[w(F_o^2)^2]\}^{1/2}$, where $w = 1/[\sigma_2(F_o^2) + (aP)^2 + bp]$, where $P = [\max(0, F_o^2) + 2F_c^2]/3$; and $Rw = [w(|F_o| - |F_c|)^2/w|F_o|^2]^{1/2}$, where $w = 1/\sigma^2(|F_o|)$.

Computational details for compound 3

The geometries and binding energies reported for both bonding motifs of **3** were computed in the same manner as that for **2**. In **3**, bonding *motif* **B**, contains two I_2 guest molecules that bind to form I_3^- units and one guest molecule that forms an $[I_4]^{2-}$ unit. The bonding energy of 21.2 kcal/mol reported for the guests that form the I_3^- units was calculated with the $[I_4]^{2-}$ guest removed.

Table S3. Experimental and DFT optimized bond distances and computed bond orders of iodine guests in two bonding motifs of compound **3**.

Iodine guest	Bond distances (Å)		Bond order
	Experimental	DFT	
I2–I19	3.41	3.38	0.25
I19–I20	2.75	2.81	0.80
I20–I10	3.47	3.42	0.21
I2B–I19B	3.33	3.14	0.45
I19B–I20B	2.74	2.79	0.76
I23–I23	2.67	2.77	0.89
I23–I10	3.97	3.89	0.15

Table S4. Assignment, occupancy and bond distances of iodine guests in compound **3**.

Iodine guest	Occupancy	Assignment	Bond distances
I13–I14	0.29	I_3^- (I3–I13–I14)	I3–I13: 3.37 Å*<br>I13–I14: 2.64 Å
I15–I16	0.30	I_3^- (I8–I15–I16)	I8–I15: 3.11 Å<br>I15–I16: 2.60 Å
I17–I18	0.22	I_3^- (I1–I17–I18)	I1–I17: 2.95 Å<br>I17-I18: 2.69 Å
I19–I20	0.69	I_4^{2-} (I2···I19–I20···I10)<br>Strongly halogen bonded	I2–I19: 3.42 Å<br>I19–I20: 2.75 Å<br>I20–I10: 3.46 Å
I19B–I20B	0.31	I_3^- (I2B–I19B–I20B)	I2B–I19B: 3.33 Å<br>I19B–I20B: 2.74 Å
I23	0.11	I_4^{2-} (I10···I23–I23···I10)<br>Weakly halogen bonded	I23–I23: 2.67 Å<br>I10–I23: 3.97 Å
I24–I25	0.35	I_4^{2-} (I11···I24–I25···I5)<br>Weakly halogen bonded	I11–I24: 4.13 Å*<br>I24–I25: 2.62 Å<br>I25-I5: 3.87 Å
I26–I27	0.30	I_3^- (I11–I26–I27)	I11–I26: 3.73 Å*<br>I26–I27: 2.54 Å

* Denoted bond distances are the average value for disordered atoms.

Description of I_2 guests in a different pore – compound 3

In addition to the I_2 guests which are part of the same pore and exhibit different binding motifs (*motifs* **A**, **B** and **C**), we observe the incorporation of three distinct I_2 guest molecules in a different section of the MOF channels. Two such guests, I13–I14 and I26–I27, are chemisorbed and form an I_3^- group. The I···I_2 interaction of I3···I13 occurs at an average distance of 3.37 Å, and is linear in fashion, giving an I3–I13–I14 angle of 176°. The analogous parameters for the I11···I26–I27 I_3^- moiety, are 3.73 Å, and 156 °. The I24–I25 molecule forms an additional $[I_4]^{2-}$ with terminal iodides I5 and

I11, yielding halogen-halogen interactions through distances of 3.87 and 4.13 Å, respectively. Lastly, I15–I16, which forms an I_3^- unit with I8, can be found in a different section than pores 1 and 2. The $I\cdots I_2$ interaction occurs through a distance of 3.11 Å, in line with other experimentally observed chemisorbed I_3^-.

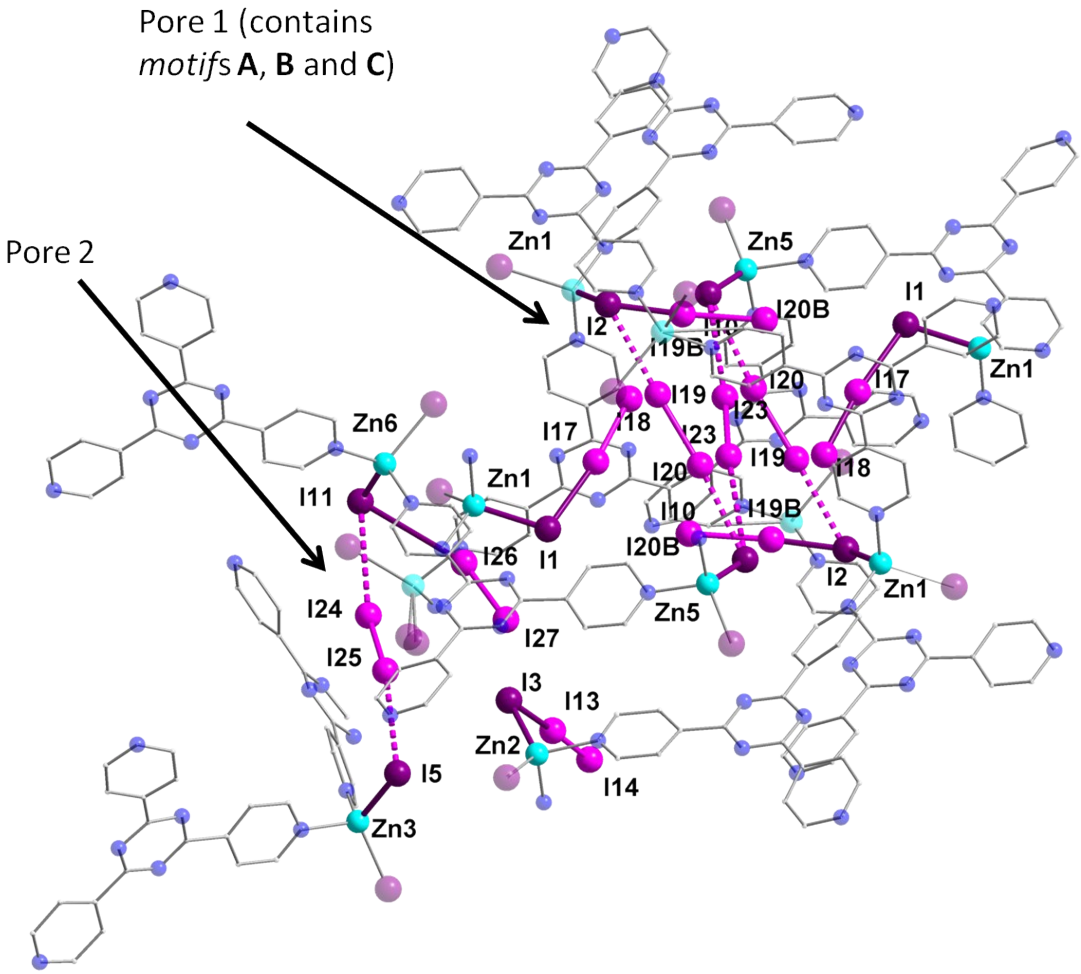

Figure S2. Partially labelled molecular structure of **3**, displaying the I_2 guests within two different parts of the MOF channels. Guest iodine atoms are shown in a lighter shade of purple than the iodine atoms belonging to the host framework. Hydrogen and disordered atoms are omitted for clarity.

TGA measurements

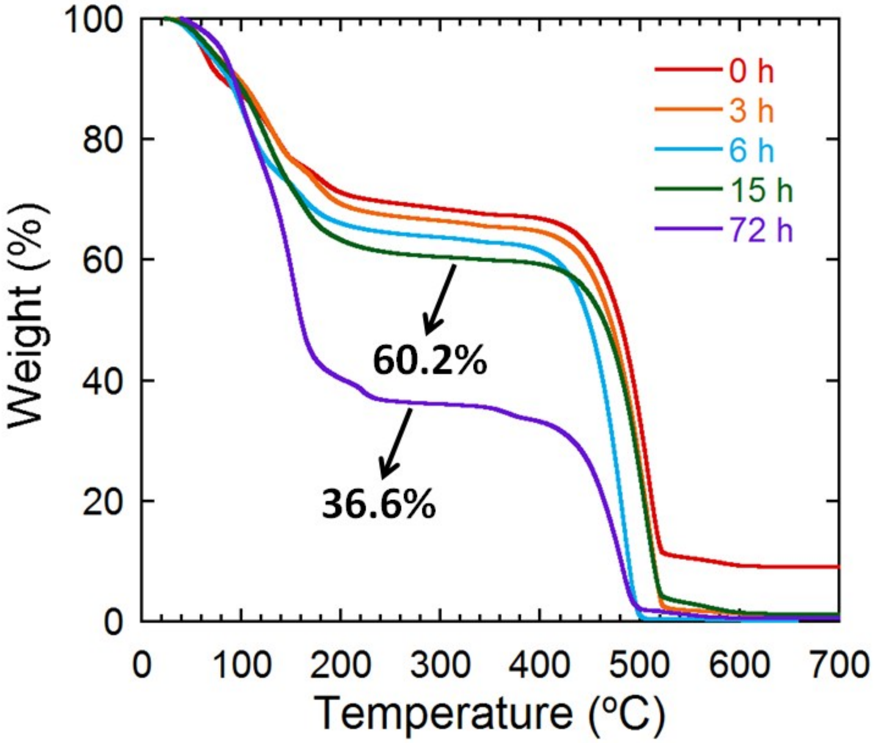

Figure S3. The thermogravimetric analyses (TGA) were carried out between 25–750 °C for **1** and between 25–1000 °C for **2**–**4** and for single crystals of **1** after 72 h of exposure to I_2. Measurements were performed under nitrogen atmosphere (25 mL/min).

The heating rate was 5 °C/min for all measurements. In all samples, the initial weight loss (25–250 °C) corresponds to the loss of guest molecules contained within the MOF. This weight loss for 0 h is due to the departure of nitrobenzene solvent molecules, while the 3 and 6 h samples have a mixture of nitrobenzene and I_2 being evacuated from the pores. As evidenced by the crystal structure of **4**, the 15 h sample does not contain any observable nitrobenzene guest molecules, and therefore, the sole contributor in this initial weight loss is uniquely I_2. The same logic can be applied to the 72 h curve. The second weight loss (375–600 °C) is attributed to the destruction of the porous solid.

Summary of I_2 guests in compound 4

Table S5. Assignment, occupancy and bond distances of iodine guests in compound **4**.

Iodine guest	Occupancy	Assignment	Bond distances
I13–I14	0.5	I_3^- (I5–I14–I13)	I5–I14: 2.92 Å*<br>I14–I13: 2.71 Å
I15–I16	0.41	I_3^- (I8–I15–I16)	I8–I15: 3.28 Å*<br>I15–I16: 2.71 Å
I17–I18	0.39	I_3^- (I4–I18–I17)	I4–I18: 3.42 Å<br>I18–I17: 2.72 Å
I19–I20	0.52	I_3^- (I3–I20–I19)	I3–I20: 3.16 Å<br>I20–I19: 2.71 Å
I21–I22	0.43	I_2 (I21–I22)	I21–I22: 2.71 Å
I23–I24	0.84	I_3^- (I10–I23–I24)	I10–I23: 3.15 Å<br>I23–I24: 2.67 Å*
I25–I26	0.83	I_3^- (I9–I26–I25)	I9–I26: 3.36 Å<br>I26–I25: 2.72 Å
I27–I28	0.77	I_4^{2-} (I6···I28–I27···I11)	I6–I28: 3.82 Å*<br>I28–I27: 2.72 Å<br>I27–I11: 3.29 Å
I29–I30	0.47	I_3^- (I12–I29–I30)	I12–I29: 3.14 Å<br>I29–I30: 2.72 Å
I31–I32	0.29	I_3^- (I6–I32–I31)	I6–I32: 3.36 Å*<br>I32–I31: 2.79 Å*
I33–I34	0.62	I_3^- (I6–I33–I34)	I6–I33: 3.38 Å<br>I33–I34: 2.71 Å
I35–I36	0.44	I_2 (I35–I36)	I35–I36: 2.72 Å
I41	0.41	I_4^{2-} (I3···I41–I41···I3)	I3–I41: 3.69 Å<br>I41–I41: 2.70 Å
I42	0.50	I_4^{2-} (I8···I42–I42···I8)	I8–I42: 3.72 Å<br>I42–I42: 2.57 Å
I43	0.80	I_4^{2-} (I1···I43–I43···I1)	I1–I43: 3.53 Å<br>I43–I43: 2.77 Å

* Denoted bond distances are the average value for disordered atoms.

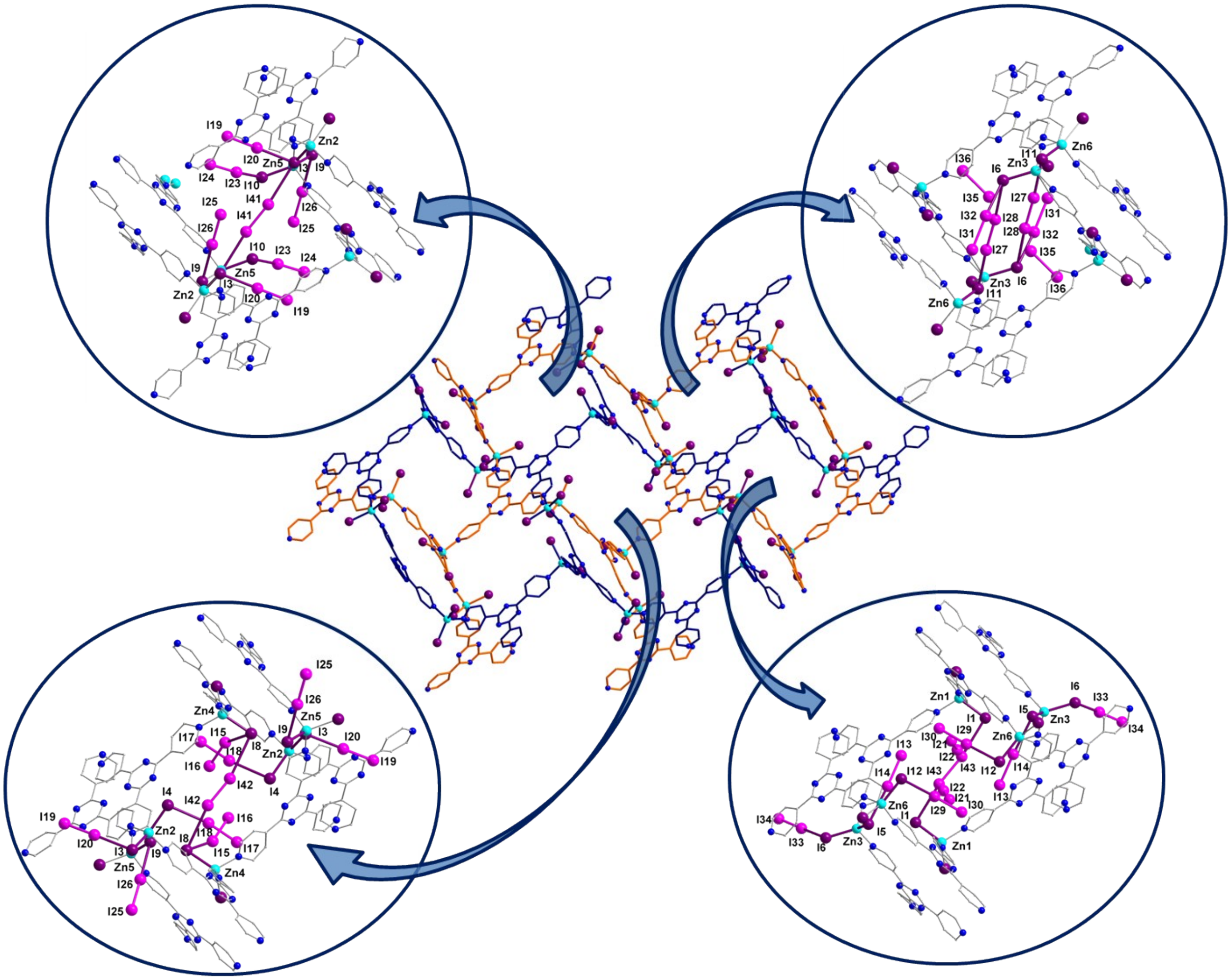

Figure S4. Packing diagram of **1** along the *b*-axis, illustrating the two interpenetrating networks in blue and orange along with the four different pores of **4** and their respective iodine guests shown in purple.

Release kinetics of I_2 and stability of the saturated compound

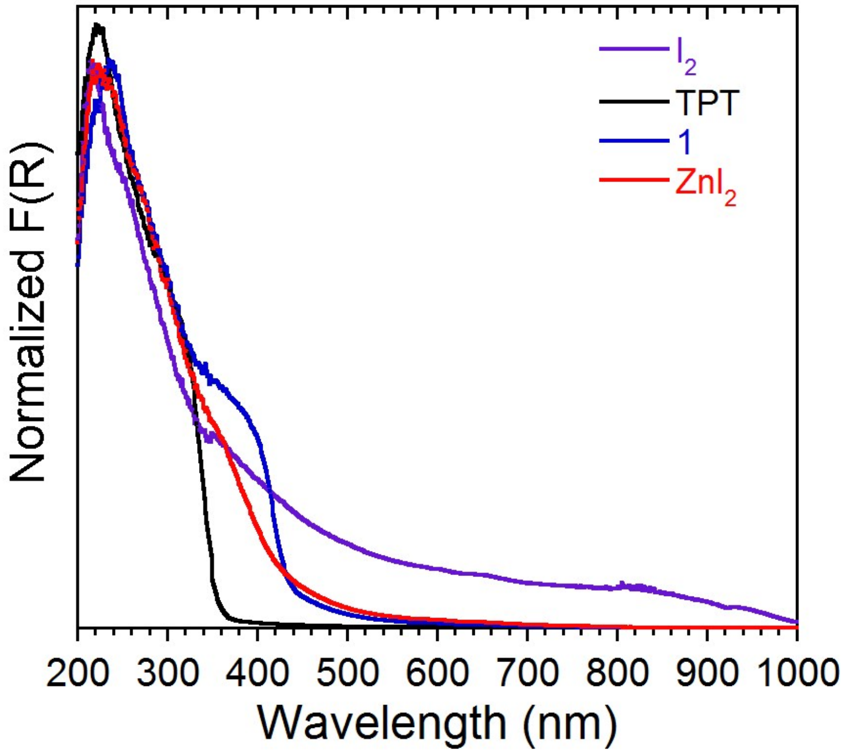

Figure S5. Normalized Kubelka-Munk spectra of TPT, ZnI_2, **1** and I_2.

The spectrum of TPT exhibits a broad band exclusively in the UV region, corresponding to intra-ligand transitions. The spectra of ZnI_2 and **1** contain an additional shoulder structure in the visible range, from 375–450 nm, most likely attributed to metal-to-ligand charge transfers (MLCT) and ligand-to-metal charge transfers (LMCT), respectively. The spectrum of I_2 displays an extremely broad band from approximately 375 to 1000 nm, corresponding to the $4\pi \rightarrow 10\sigma^*$ electronic transition.

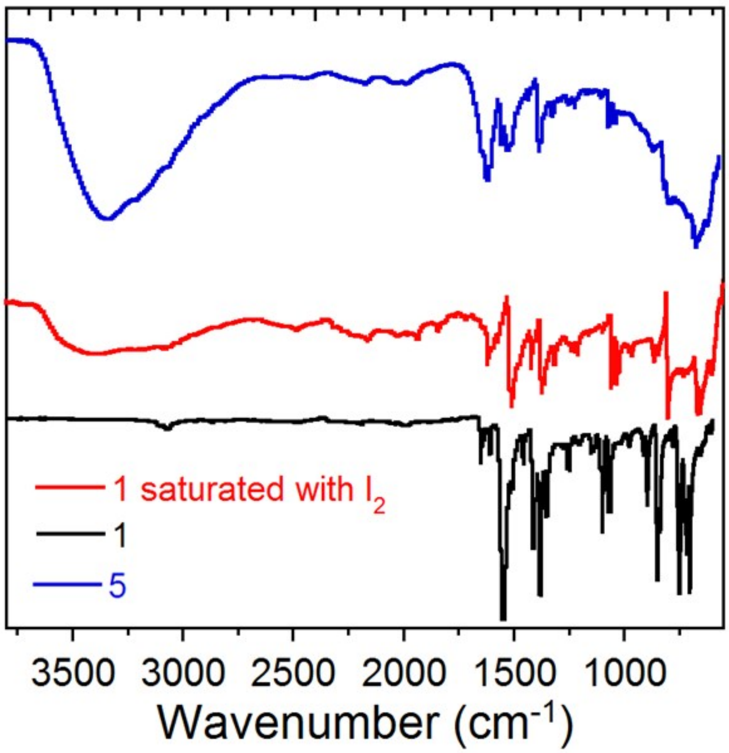

Figure S6. FTIR spectra of **1**, **1** completely saturated by I_2 and **1** saturated by I_2 after 96 hours at ambient conditions (**5**).

Description of short halogen contacts

Short halogen atom contacts in crystals have recently grown to be one of the most interesting noncovalent interactions for constructing supramolecular assemblies.[12] These interactions include halogen···halogen (X···X) and halogen···heteroatom (X···B) interactions. It was established that R−X···X−R contacts occur in two preferential geometries, which were classified as type I (symmetrical interactions where $\theta_1 = \theta_2$) and type II (bent interactions where $\theta_1 \approx 180°$ and $\theta_2 \approx 90°$) (Fig. S7).[13] There is a clear geometric and chemical distinction between type I and type II X···X interactions. Type I interactions are geometry-based contacts that arise from close-packing requirements and are found for all halogens. Interestingly, such interactions are not halogen bonds

according to the IUPAC definition.[14] Type II interactions arise from the pairing between the electrophilic area on one halogen atom and the electrophilic area on the other, and consist of true halogen bonds.[15] It was found that type II contacts, being selective and directional, as well as having energies comparable to that of hydrogen bonds[16] are most favored in iodinated derivatives, less in brominated derivatives, and the least in chlorinated derivatives.[15]

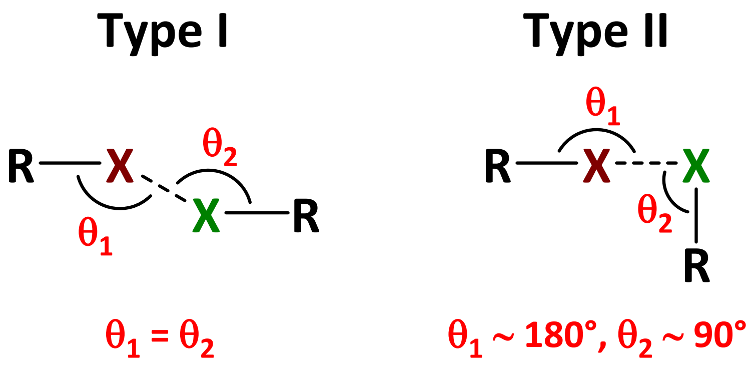

Figure S7. Structural scheme for type I and type II halogen···halogen short contacts.

For example, in the structure of **2**, the Zn3–I5···I22 and Zn4–I7···I21 bond angles are about 96.13° and 115.76°, respectively. This, together with almost linear I15···I22–I21 and I7···I21–I22 angles and short I5···I22 and I7···I21 separations, testify to the presence of remarkably strong dihalogen interactions.

Supplemental references

(1) M.-X. Li, Z.-X. Miao, M. Shao, S.-W. Liang, S.-R. Zhu, *Inorg. Chem.*, 2008, **47**, 4481.

(2) K. Biradha, M. Fujita, *Angew. Chem. Int. Ed.*, 2002, **41**, 3542.

(3) A. L. Speck, *Acta Cryst.*, 2009, **D65**, 148.

(4) G. M. Sheldrick, *Acta Cryst.*, 2008, **A64**, 112.

(5) (*a*) G. Kresse, J. Furthmüller, *J. Comput. Mater. Sci.*, 1996, **6**, 15; (*b*) G. Kresse, J. Hafner, *J. Phys. Rev. B*, 1993, **47**, 558; (*c*) G. Kresse, J. Hafner, *J. Phys. Rev. B*, 1994, **49**, 14251; (*d*) G. Kresse, J. Furthmüller, *J. Phys. Rev. B*, 1996, **54**, 11169.

(6) J. P. Perdew, K. Burke, M. Ernzerhof, *Phys. Rev. Lett.,* 1996, **77**, 3865.

(7) P. E. Blöchl, *Phys. Rev. B*, 1994, **50**, 17953.

(8) G. Kresse, *Phys. Rev. B*, 1999, **59**, 1758.

(9) S. Grimme, *J. Comput. Chem.*, 2006, **27**, 1787.

(10) M. J. Frisch, G. W. Trucks, H. B. Schlegel, G. E. Scuseria, M. A. Robb, J. R. Cheeseman, G. Scalmani, V. Barone, B. Mennucci, G. A. Petersson, H. Nakatsuji, *Gaussian 09, Revision C.01* (Gaussian, 2010).

(11) (*a*) J. K. Badenhoop, F. Weinhold, *J. Chem. Phys.*, 1997, **107**, 5406; (*b*) E. D. Glendening, C. R. Landis, F. Weinhold, *J. Comput. Chem.*, 2013*,* **34**, 1429; (*c*) F. Weinhold, C. R. Landis, *Valency and Bonding: A Natural Bond Orbital Donor–Acceptor Perspective* (Cambridge Univ. Press, 2005). (*d*) A. E. Reed, L. A. Curtiss, F. Weinhold, *Chem. Rev.*, 1988, **88**, 899.

(12) P. Metrangolo, G. Resnati, *Halogen Bonding: Fundamentals and Applications (Structure and Bonding)* (Springer, Heidelberg, 2010).

(13) G. R. Desiraju, R. Parthasarathy, *J. Am. Chem. Soc.*, 1989, **111**, 8725.

(14) G. R. Desiraju, P. S. Ho, L. Kloo, A. C. Legon, R. Marquardt, P. Metrangolo, P. Politzer, G. Resnati, K. Rissanen, *Pure Appl. Chem.*, 2013, **85**, 1711.

(15) A. Mukherjee, S. Tothadi, G. R. Desiraju, *Acc. Chem. Res.*, 2014, **47**, 2514.

(16) C. B. Aakeröy, M. Fasulo, N. Schultheiss, J. Desper, C. Moore, *J. Am. Chem. Soc.*, 2007, **129**, 13772.
